# Altered microRNA expression in frontotemporal lobar degeneration with TDP-43 pathology caused by progranulin mutations

**DOI:** 10.1186/1471-2164-12-527

**Published:** 2011-10-27

**Authors:** Jannet Kocerha, Naomi Kouri, Matt Baker, NiCole Finch, Mariely DeJesus-Hernandez, John Gonzalez, Kumaravel Chidamparam, Keith A Josephs, Bradley F Boeve, Neill R Graff-Radford, Julia Crook, Dennis W Dickson, Rosa Rademakers

**Affiliations:** 1 Department of Neuroscience, Mayo Clinic College of Medicine, Jacksonville, FL 32224, USA; 2 Ocean Ridge Biosciences, 10475 Riverside Drive, Suite 1, Palm Beach Gardens, FL 33410, USA; 3 Department of Neurology, Mayo Clinic College of Medicine, Rochester, MN, 55905, USA; 4 Department of Neurology, Mayo Clinic College of Medicine, Jacksonville, FL 32224, USA

**Keywords:** Frontotemporal lobar degeneration, TDP-43, microRNA, progranulin

## Abstract

**Background:**

Frontotemporal lobar degeneration (FTLD) is a progressive neurodegenerative disorder that can be triggered through genetic or sporadic mechanisms. MicroRNAs (miRNAs) have become a major therapeutic focus as their pervasive expression and powerful regulatory roles in disease pathogenesis become increasingly apparent. Here we examine the role of miRNAs in FTLD patients with TAR DNA-binding protein 43 pathology (FTLD-TDP) caused by genetic mutations in the progranulin (*PGRN*) gene.

**Results:**

Using miRNA array profiling, we identified the 20 miRNAs that showed greatest evidence (unadjusted P < 0.05) of dysregulation in frontal cortex of eight FTLD-TDP patients carrying *PGRN *mutations when compared to 32 FTLD-TDP patients with no apparent genetic abnormalities. Quantitative real-time PCR (qRT-PCR) analyses provided technical validation of the differential expression for 9 of the 20 miRNAs in frontal cortex. Additional qRT-PCR analyses showed that 5 out of 9 miRNAs (miR-922, miR-516a-3p, miR-571, miR-548b-5p, and miR-548c-5p) were also significantly dysregulated (unadjusted P < 0.05) in cerebellar tissue samples of *PGRN *mutation carriers, consistent with a systemic reduction in PGRN levels. We developed a list of gene targets for the 5 candidate miRNAs and found 18 genes dysregulated in a reported FTLD mRNA study to exhibit anti-correlated miRNA-mRNA patterns in affected cortex and cerebellar tissue. Among the targets is brain-specific angiogenesis inhibitor 3, which was recently identified as an important player in synapse biology.

**Conclusions:**

Our study suggests that miRNAs may contribute to the pathogenesis of FTLD-TDP caused by *PGRN *mutations and provides new insight into potential future therapeutic options.

## Background

Frontotemporal lobar degeneration (FTLD) is the second most common cause of early-onset dementia after Alzheimer's Disease (AD) [1]. FTLD patients are clinically characterized by personality changes and disinhibited behaviour, often combined with a gradual and progressive language dysfunction [2]. Memory impairment is typically preserved in the early phase of disease, which distinguishes them from patients with AD. Pathologically, around 40% of FTLD patients present with neuronal and/or glial tau aggregates (FTLD-tau), whereas the majority of FTLD patients show ubiquitin-immunoreactive cytoplasmic and intranuclear inclusions historically referred to as FTLD-U (FTLD with ubiquitin-positive inclusions) [3]. More recently, it was shown that hyperphosphorylated and C-terminal truncated fragments of the nuclear protein TAR DNA-binding protein 43 (TDP-43) were the main component of the pathological inclusions in FTLD-U, and the term FTLD-TDP was introduced [4,5]. Three main patterns of TDP-43 pathology are recognized in FTLD-TDP, based on the anatomical distribution, morphology, and relative proportion of distinct types of inclusions [6,7]. In this study, we will follow the nomenclature based on the Mackenzie scheme [6] where FTLD-TDP type 1 is characterized by TDP-43 positive compact neuronal cytoplasmic inclusions and short neurites, FTLD-TDP type 2 presents with long TDP-43 positive neurites and FTLD-TDP type 3 is characterized by compact and granular cytoplasmic inclusions.

In the past decade, several different genes and chromosomal loci have been associated with FTLD. Mutations in the microtubule associated protein tau (*MAPT*) gene were first identified as a cause of familial FTLD-tau [8-10]. More recently, our group and others discovered that heterozygous mutations in the progranulin gene (*PGRN*) cause FTLD-TDP through a loss of function mechanism [11,12]. Patients with *PGRN *mutations maintain only a single functional copy of the gene, leading to the loss of 50% of functional *PGRN*, causing disease through haploinsufficiency. The reduced level of *PGRN*, a growth factor with a key role in a variety of cellular responses, provokes neurodegeneration and associated symptomatology in FTLD patients, including deficits in behaviour, language, and movement [13-15]. Interestingly, all patients with *PGRN *mutations present with FTLD-TDP pathology type 1 [16,17]; however, FTLD-TDP Type 1 is also observed in a subset of FTLD-TDP patients without *PGRN *mutations. Although there are clear pathologic distinctions in FTLD-TDP, the molecular pathways which underlie its progression are still mostly undefined. Recent advances in our understanding of the mammalian genomes, however, have revealed novel regulatory mechanisms with critical roles in disease pathogenesis, thus offering new avenues to explore.

The recent discovery of pervasive expression for noncoding RNAs (ncRNAs) in our genomes through extensive 'transcriptomic' efforts [18-23] has significantly enhanced our fundamental knowledge of cellular signaling cascades and will likely reshape future drug discovery efforts. Indeed, *PGRN *mutation carriers diagnosed with FTLD exhibit a range of pathologic and phenotypic outcomes, suggesting that other contributing factors, such as ncRNAs, mediate disease progression [4,11,12,15,19,24].

The miRNA class of ncRNA, in particular, has generated a lot of interest as their widespread role in many cellular functions becomes increasingly apparent [18,25-31]. One miRNA can control the expression of hundreds of downstream gene targets, underscoring the importance of characterizing their functional roles *in vitro *and *in vivo *[25]. Over the last few years, a growing number of publications have reported dysregulation of miRNA expression in numerous diseases, including neurodegenerative disorders, such as Alzheimer's disease and Huntington's disease [28,32-34]. Recent reports have also examined miRNA regulation of *PGRN*, suggesting that this gene is under the control of ncRNAs, including miR-107, and miR-29b [35,36]. Furthermore, our group previously showed a functional disruption of a miR-659 binding site in FTLD patients with a common genetic variant of *PGRN *(rs5848) [37].

Here we profiled miRNA expression in the frontal cortex of a population of FTLD-TDP patients with *PGRN *mutations and compared their miRNA expression pattern with a large group of FTLD-TDP patients without *PGRN *mutations, with the goal to identify miRNAs responsive to *PGRN *haploinsufficiency. For those miRNAs showing greatest evidence of dysregulation and that were validated technically by quantitative real-time PCR in frontal cortex, we further examined their expression in the cerebellum, with the expectation that PGRN levels are globally disrupted throughout the CNS. Finally, we developed a unique list of gene targets predicted to be regulated by miRNAs dysregulated in both frontal cortex and cerebellum in our patient samples, based on previously reported microarray mRNA data as well as bioinformatic miRNA target prediction sites.

## Results

### miRNA array results from frontal cortex

**Figure 1:**
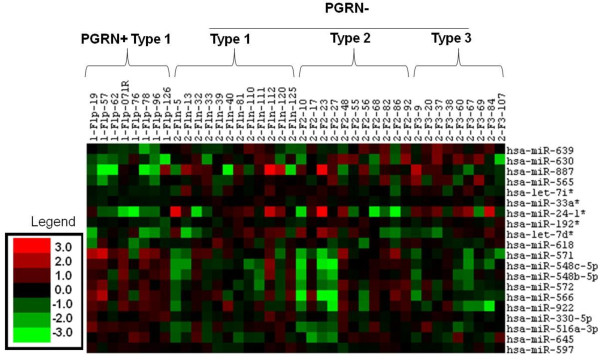
**Cluster diagram of Ct values for the 20 significant miRNAs from the array profiling**. Each column represents a single sample, and each row represents a single miRNA. Green squares represent lower than median level of miRNA expression; black squares represent median level of miRNA expression; red squares represent higher than median level of miRNA expression. Legend units: 1.0 = differs from median probe intensity by one Ct unit. In addition to *PGRN*+ and *PGRN*- FTLD-TDP patients, 3 different FTLD-TDP pathologic subtypes are represented in this heat map: *PGRN*+ FTLD-TDP type 1 and *PGRN*- FTLD-TDP types 1, 2, and 3.

Using the ABI v2.0 arrays, we profiled the expression of 664 miRNAs from 40 FTLD-TDP patients, including 32 *PGRN*- FTLD-TDP and 8 *PGRN*+ FTLD-TDP patients (patient demographics listed in Table 1). There was detectable expression for 490 of the 664 miRNAs present on the array, and those candidate miRNAs were subjected to further analysis. We identified the 20 miRNAs which showed greatest evidence of differential expression (those with unadjusted P < 0.05) between the *PGRN+ *and *PGRN- *FTLD-TDP groups (Figure 1). None of these were statistically significant after accounting for multiple testing (overall P = 0.34) and the smallest q-value was 0.57, however we still pursued these 20 top-ranking miRNAs as the most promising candidates. Ten out of the 20 miRNAs had higher expression in the *PGRN+ *FTLD-TDP group (miR-571, miR548c-5p, miR-572, miR-922, miR-516a-3p, miR-566, miR-597, miR-645, miR-548b-5p, miR330-5p), while the other 10 miRNAs had lower expression in this group (miR-33a*, miR639, miR-630, miR-887, miR-565, miR-192*, miR-618, miR-24-1*, let-7d*, and let-7i*). All miRNA array results and statistical analyses are listed in Additional File 1. Graphical display of the 20 significantly changed miRNAs showed a consistent miRNA expression pattern in all three *PGRN*- FTLD-TDP subtypes (type 1, 2 and 3) compared to *PGRN*+ FTLD-TDP (Additional File 2).

**Table 1:** Patient demographics for frontal cortex and cerebellar brain samples included in this study.

Mutation status	N	FTLD-TDP Type 1	FTLD-TDP Type 2	FTLD-TDP Type 3	Male/Female	Mean age at death ± SD
***PGRN *+ Cortex**	8	8	n/a	n/a	3/5	75.5 ± 10.1 (63-87)

***PGRN *-** **Cortex**	32	12	11	9	18/14	74.9 ± 10.5 (52-97)

***PGRN *+** **Cerebellum**	10	10	n/a	n/a	7/3	72.3 ± 10.3 (56-87)

***PGRN *-** **Cerebellum**	30	12	12	6	16/14	73.8 ± 10.3 (52-97)

* There was overlap of 6 PGRN+ and 25 PGRN- samples between the cerebellum and frontal cortex analyses. n/a = not applicable

### miRNA validation from frontal cortex

**Figure 2:**
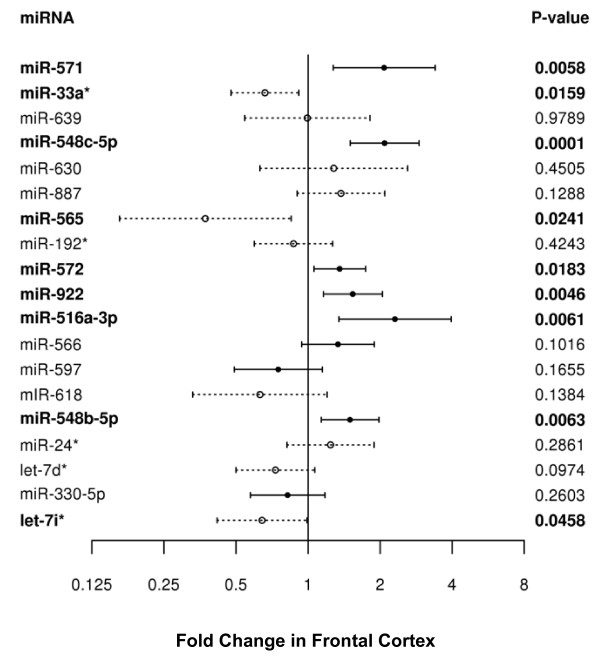
**Validation of miRNA candidates in frontal cortex of FTLD patients**. For each miRNA, fold change of *PGRN*+ FTLD-TDP patients compared to *PGRN*- FTLD-TDP patients with 95% confidence intervals are shown with their associated p-value. Significantly dysregulated miRNAs are shown in bold. Solid bars represent miRNAs which were upregulated in the original miRNA array, dashed bars represent miRNAs downregulated in the original miRNA array.

To perform technical validation of the miRNA array results, we evaluated the expression of the 20 significantly dysregulated miRNAs between the *PGRN+ *and *PGRN- *FTLD-TDP patients by qRT-PCR. One miRNA, miR-645, was undetectable using the individual miRNA assays from ABI. Of the remaining 19 detectable miRNAs, nine could be confirmed by qRT-PCR as significantly altered between the *PGRN+ *and *PGRN- *FTLD-TDP groups with P < 0.05. The validated miRNAs were miR-565, miR-33a*, let7i*, miR-922, miR-571, miR-572, miR-548b-5p, miR-548c-5p and miR-516a-3p (Figure 2).

### miRNA validation from cerebellum

**Figure 3:**
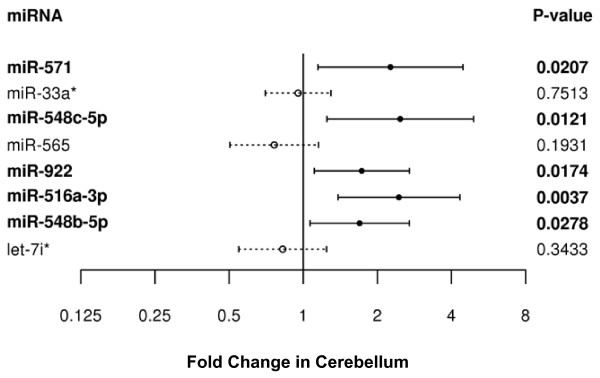
**Validation of miRNA candidates in cerebellum of FTLD patients**. For each miRNA, fold change of *PGRN*+ FTLD-TDP patients compared to *PGRN*- FTLD-TDP patients with 95% confidence intervals are shown with their associated p-value. Significantly dysregulated miRNAs are shown in bold. Solid bars represent miRNAs which were upregulated in the frontal cortex, dashed bars represent miRNAs downregulated in the frontal cortex.

We hypothesized that the miRNAs dysregulated in the frontal cortex of the *PGRN+ *FTLD-TDP patients could also be differentially expressed in other brain areas as haploinsufficiency of *PGRN *function would not be region-specific. We therefore selected the 8 frontal cortex validated miRNAs with the largest estimated fold change and profiled their expression in the cerebellum of 10 *PGRN*+ FTLD-TDP and 30 *PGRN*- FTLD-TDP patients (patient demographics listed in Table 1, 31 patients common to frontal cortex analyses). Five of the 9 miRNAs (miR-922, miR-516a-3p, miR-571, miR-548b-5p, and miR-548c-5p), were significantly altered with P < 0.05 in the cerebellum (Figure 3).

### miRNA expression *in vitro*

To further study the relationship between the loss of PGRN and the top five dysregulated miRNAs identified in frontal cortex and cerebellum, we performed a preliminary *in vitro *study in human neuroblastoma SH-SY5Y cells. In cells treated with PGRN siRNA we detected a 60% decrease in *PGRN *mRNA levels compared to negative control siRNA treated cells; however, no difference in miRNA expression for miR-516a-3p, miR-548b-5p and miR-548c-5p was observed (Additional File 3). Expression of miR-571 and miR-922 was too low (Ct < 35) and could not be included in the analysis.

### TargetScan analysis and Ingenuity pathway analysis of miRNA targets

The overall role of miRNAs is to repress mRNA and protein expression [25,38]. To facilitate the identification of mRNA targets for the 5 *PGRN+ *FTLD-TDP associated miRNAs, we made use of publicly available Affymetrix mRNA arrays performed in FTLD-TDP patients with and without *PGRN *mutations (GEO record GDS3459) [39]. Since all 5 miRNAs were upregulated in frontal cortex and cerebellum of *PGRN *mutation carriers, we focused on mRNA targets which were downregulated in both frontal cortex and cerebellum of *PGRN *mutations carriers in the Affymetrix mRNA arrays. A total of 177 probesets showed significant downregulated expression in both the cortex and cerebellum of the *PGRN+ *FTLD-TDP patients (P < 0.05). When compared with the list of TargetScan predicted genes for each of the 5 *PGRN*+ FTLD-TDP associated miRNAs, 18 genes with anti-correlated mRNA-miRNA expression were identified (Table 2). Among the 18 genes, brain-specific angiogenesis inhibitor 3 (BAI3), glycerol kinase (GK) and solute carrier family 23, member 2 (SLC23A2) were targeted by 3 of the 5 miRNAs upregulated in the cortex and cerebellum of the *PGRN+ *FTLD-TDP patients. Seven genes were targeted by 2 of the 5 miRNAs, and 8 genes were targeted by 1 of the 5 miRNAs (Table 2).

**Table 2:** Candidate gene targets identified in this study.

Gene	Name	miRNA species
**BAI3**	brain-specific angiogenesis inhibitor 3	miR-922; miR-548b-5p; miR-548c-5p

**GK**	glycerol kinase	miR-571; miR-548b-5p; miR-548c-5p

**SLC23A2**	solute carrier family 23 (nucleobase transporters), member 2	miR-922; miR-548b-5p; miR-548c-5p

**GTDC1**	glycosyltransferase-like domain containing 1	miR-516a-3p; miR-571

**CNR1**	cannabinoid receptor 1	miR-548b-5p; miR-548c-5p

**FAM134B**	family with sequence similarity 134, member B	miR-548b-5p; miR-548c-5p

**HS2ST1**	heparan sulfate 2-O-sulfotransferase 1	miR-548b-5p; miR-548c-5p

**MYT1L**	myelin transcription factor 1-like	miR-548b-5p; miR-548c-5p

**NCAM1**	neural cell adhesion molecule 1	miR-548b-5p; miR-548c-5p

**TMEM135**	transmembrane protein 135	miR-548b-5p; miR-548c-5p

**ATP8A1**	ATPase, class I, type 8A, member 1	miR-922

**KCNAB1**	potassium voltage-gated channel, shaker-related subfamily, beta member 1	miR-922

**PTPRD**	protein tyrosine phosphatase, receptor type, D	miR-922

**RASA1**	RAS p21 protein activator 1	miR-922

**REEP1**	receptor accessory protein 1	miR-922

**SNCA**	synuclein, alpha	miR-922

**A2BP1**	ataxin-2-binding protein 1	miR-516a-3p

**ASTN1**	astrotactin 1	miR-516a-3p

List of top scoring genes predicted to be regulated by the 5 significantly upregulated miRNAs (miR-922, miR-516a-3p, miR-571, miR-548b-5p, and miR-548c-5p) from the 177 mRNAs significantly downregulated in frontal cortex and cerebellum in *PGRN+ *compared to PGRN- FTLD-TDP patients (GEO record GDS3459).

Next, for the 18 genes we found in common between the TargetScan and Affymetrix results, we examined their potential biological roles with Ingenuity Pathway analysis. Interestingly, neurological and cellular regulations were the most prominently represented biological roles of the significant pathways identified (P value < 0.05). In fact, 6 of the genes (astrotactin 1 (*ASTN1*), protein tyrosine phosphatase, receptor type, D *(PTPRD)*, potassium voltage-gated channel, shaker-related subfamily, beta member 1 (*KCNAB1*), cannabinoid receptor 1 (*CNR1*), alpha-synuclein (*SNCA*), and neural cell adhesion molecule 1 (*NCAM1*)) were shown to have a specific role in behavioural responses, a phenotype which is consistently altered in FTLD (Additional File 4).

## Discussion

Identifying the molecular events leading to pathogenic outcomes in neurodegenerative diseases, such as FTLD, may ultimately produce new avenues for prevention or treatment of these disorders. In this study, we report a novel role for ncRNAs in the molecular profile of FTLD patients with genetic mutations in the secreted growth factor PGRN. The miRNA family of ncRNAs showed distinct expression patterns in post-mortem brain tissue of FTLD-TDP patients carrying loss-of function mutations in *PGRN *compared to FTLD-TDP patients without known mutations, suggesting that miRNAs are potential biomarkers and therapeutic targets for genetically-linked dementia disorders.

Since the initial reports linking *PGRN *mutations to FTLD [11,12], the search for *PGRN *mediated signaling cascades has intensified, such as the recently reported associations with sortilin (SORT1) [40,41]. Through the comparison of ncRNA expression profiles from patients with genetic versus non-genetic (or sporadic) diagnosis of FTLD-TDP, we aimed to identify new pathways which are under the control of *PGRN *signaling *in vivo*. Indeed, in frontal cortex samples of FTLD-TDP patients, the expression of 3% of the detectable miRNAs from the expression arrays was significantly changed when *PGRN*+ and *PGRN*- FTLD-TDP patients were compared. Moreover, the dysregulated miRNAs showed consistent trends in all three of the *PGRN*- FTLD-TDP subtypes (types 1-3) compared to *PGRN*+ FTLD-TDP patients, suggesting the miRNA candidates we identified are unique to *PGRN *haploinsufficiency. In further support that the miRNAs dysregulated in our array and validation studies are under the control of systemic *PGRN*-mediated mechanisms, we found that 5 miRNAs (miR-548b-5p, miR-548c-5p, miR-571, miR-922, and miR-516a-3p) were also upregulated in the cerebellum of *PGRN+ *FTLD-TDP compared to *PGRN*- FTLD-TDP patients.

To further study the five candidate miRNAs, we silenced *PGRN *expression in SH-SY5Y cells; however, none of the 3 miRNAs detectable in SY-SY5Y cells displayed a significant difference in expression between control and PGRN-silenced cells. This finding suggests that long-term knockdown of *PGRN *may be necessary, consistent with the late onset of symptoms in human FTLD patients.

The mechanism by which *PGRN *haploinsufficiency in FTLD patients leads to altered miRNA expression is currently unclear and requires future studies. Progranulin downstream signalling involves ERK1/2 and AKT signalling and these are potential causes of altered miRNA expression. It is unlikely that the five miRNAs identified in this study are dysregulated as a result of TDP-43 aggregation since the FTLD-TDP type 1 pathology in the *PGRN *mutation carriers is indistinguishable from the pathology observed in sporadic FTLD-TDP patients.

It is now known that miRNAs can modulate mRNA stability and translation [25,38], therefore, we correlated publicly available mRNA expression results from sporadic (*PGRN*-) FTLD-TDP and *PGRN+ *FTLD-TDP patients with bioinformatic miRNA target predictions (TargetScan) for the 5 miRNAs upregulated in the frontal cortex and cerebellum [39]. Through this analysis, we identified 18 predicted gene targets with significantly downregulated mRNA expression profiles in *PGRN+ *FTLD-TDP patients. The anti-correlated expression of the upregulated miRNAs with their downregulated mRNA targets in *PGRN+ *patients parallels the established miRNA-mRNA regulatory relationship. Notably, Ingenuity pathway analysis of the 18 genes revealed that they have important links to biological functions involved in FTLD disease pathogenesis, including nervous system development, behavioural responses, and cell growth. Indeed, *ASTN1 *is known to regulate neuronal migration in cortical regions of developing brain [42], *SNCA *is associated with neurodegeneration and dementias, including links to FTLD-TDP in *PGRN*+ patients [43-46] and *REEP1 *has been implicated in corticospinal neurodegenerative disorders [47]. Importantly, only 3 genes are predicted to be targeted by 3 of the 5 miRNAs significantly dysregulated in both frontal cortex and cerebellum, including *BAI3*, a cell adhesion G protein coupled receptor. This finding is of significant interest since Bolliger *et al*. recently reported that C1q-like proteins can act as secreted signalling molecules that bind to BAI3 leading to the regulation of synapse formation and maintenance. In support of their finding, both C1ql and BAI3 are highly and specifically expressed in brain and are enriched in neurons [48]. Based on these findings our current data suggest that the loss of PGRN may increase the expression of miR-922, miR-548b-5p and miR-548c-5p through unknown mechanisms, leading to a decrease in the levels of BAI3, an essential protein for synapse biology.

## Conclusions

Overall, our studies support a novel role for miRNAs in FTLD-TDP due to *PGRN *dysfunction and emphasize the value of combined miRNA and mRNA analyses. Future experiments in cell and animal models are needed to further evaluate the clinical potential of the miRNAs and gene targets identified in this study. The recent progress in human trials for miRNA-based therapeutics in non-CNS related disorders [49,50] offers hope for new alternatives for the treatment of dementias, including FTLD.

## Methods

### Brain samples

For the miRNA array experiment, post-mortem midfrontal cortex tissue was isolated from a collection of 40 FTLD-TDP patients selected from the Mayo Clinic Jacksonville (MCJ) brain bank. All samples were obtained with appropriate informed consent with ethical committee approval. FTLD patients included the following pathologic classifications: FTLD-TDP type 1 without *PGRN *mutations (*PGRN*- type 1, N = 12), FTLD-TDP type 2 (N = 11), FTLD-TDP type 3 (N = 9) and FTLD-TDP type 1 with *PGRN *mutations (*PGRN*+ type I, N = 8). Total RNA quantification was performed using a NanoDrop ND-1000 spectrophotometer (NanoDrop, Wilmington, DE). RNA quality was evaluated by the Agilent RNA 6000 Nano Kit (Agilent, Santa Clara, CA) and only samples with an RNA integrity value greater than 5 were included in this study. Mean RINs in frontal cortex were *PGRN*+ (5.6 ± 0.4), *PGRN*- type 1 (6.2 ± 0.6), FTLD-TDP type 2 (6.2 ± 1.0), and FTLD-TDP type 3 (6.4 ± 0.8). Mean RINs in cerebellums were *PGRN*+ (7.1 ± 0.6), *PGRN*- type 1 (7.0 ± 1.9), FTLD-TDP type 2 (7.2 ± 1.8), and FTLD-TDP type 3 (7.9 ± 0.4). Cerebellar tissue of sufficient quality for miRNA expression analyses was also available for 31 of these FTLD-TDP patients. For the miRNA expression analyses in cerebellum, 9 additional FTLD-TDP patients were obtained from the MCJ brain bank. Importantly, all *PGRN *mutations included in this study were clear pathogenic loss-of-function mutations, leading to haploinsufficiency. Demographic and neuropathologic information on all patients included in this study are summarized in Table 1.

### miRNA array analyses

For mature miRNA expression profiling, real-time RT-PCR was performed using TaqMan Human MicroRNA Low Density Arrays Version 2.0 (Applied Biosystems) which contain 667 unique assays specific to human mature miRNAs in a two-card format. Total RNA was isolated from human frontal cortical tissue using the miRVana PARIS kit from Ambion (Applied Biosystems). Total RNA (150 ng) was reverse transcribed to cDNA for mature miRNAs using Megaplex RT Primers (Applied Biosystems) in 7.5 μls of final reaction volume. Subsequently, 2.5 μls of cDNA was pre-amplified in a 25 μl final volume with PreAmp Master Mix and Megaplex PreAmp Primers using standard conditions according to manufacturer's instructions. Preamplified cDNA was diluted in 0.1× Tris-EDTA (pH 8.0), applied to miRNA real-time array plates and mature miRNA expression was assessed using Applied Biosystems 7900HT System.

### Statistical analysis of miRNA array data

There were 664 miRNAs profiled for each of the 40 samples; Ct values were obtained with the automatic baseline and manual Ct set to 0.1 threshold. Some miRNAs were only minimally expressed, and were excluded from further analyses; specifically we excluded those for which 20% or more of the samples had a missing Ct or Ct > 35. Lowess smoothing was used to normalize measures across individuals. Missing values were imputed using a K-Nearest Neighbour approach as described by Tusher et al. [51]. Any particularly extreme values for each miRNA were 'shrunk' in towards the center of the distribution so as to lessen their influence. For each comparison of two groups, two sample t-tests were used to assess nominal significance. The Westfall-Young min-P approach using 1000 permutations of group labels was used to obtain p-values adjusted for multiple testing [52]. Empirical q-values were also estimated using the permuted data.

### Heat Maps and Box plots based on the miRNA array data

Normalized Ct values were adjusted by subtracting the Ct value from an arbitrary constant value of 40 so that a higher adjusted Ct value would correspond to a higher miRNA expression. The table of adjusted Ct values for the 20 significantly dysregulated miRNAs (P < 0.05) between *PGRN*+ and *PGRN*- FTLD-TDP patients was loaded in Cluster 3.0 [53]. A heat map showing the miRNA expression profiles for all the samples was generated after median centering the adjusted Ct values for each miRNA. The normalized and adjusted Ct values were summarized across groups with boxplots.

### Validation of miRNA candidates in frontal cortex and cerebellum

The top 20 miRNA candidates identified in the miRNA array experiment were selected for validation by qRT-PCR in the same set of 8 *PGRN*+ and 32 *PGRN*- FTLD-TDP patient samples. In brief, 50 μls of reverse transcription primers (miRNA assay kits from ABI) for the 20 miRNAs plus RNU48 as an endogenous control were divided into 3 primer pools, lyophilized, and subsequently resuspended in water for each pool resulting in 5× multiplex RT primer pool. Total RNA (200 ng) was reverse transcribed in a 20-μl reaction volume using the miRNA Reverse Transcription Kit (Applied Biosystems) and 1 μl of cDNA was used in the Taqman miRNA assays [19]. Where duplicate Ct values differed by more than 2, the more extreme one relative to the distribution of Ct values across all samples was deleted; otherwise the mean of the duplicates was used as the final Ct for a transcript. Delta Cts were calculated by subtracting the Ct of the endogenous control RNU48. Minus delta Cts were used as the final values for analysis and assumed to represent the log base 2 of scaled expression levels. Two sample t-tests and corresponding 95% confidence intervals (CI) were used to compare groups, and the differences between means and CIs were exponentiated to provide fold change estimates under the assumption of perfect probe efficiency.

For a total of 8 miRNAs validated in frontal cortex and showing the largest fold-change, we also performed qRT-PCR using cerebellum tissue samples from 10 *PGRN*+ and 30 *PGRN*- FTLD-TDP patients, using similar techniques and analyses, as described above. Individual Ct values in the validation data sets for both the cortex and cerebellum are provided in Additional File 5.

### miRNA expression *in vitro*

SH-SY5Y cells were cultured in a 1:1 mixture of OPTI-MEM and DMEM containing 5% heat-inactivated FBS and 1% penicillin/streptomycin. Cell cultures were kept at 37°C in a humidified atmosphere with 5% CO_2_. Cells were seeded at 175, 000 cells/well in 6-well plates and 24 h later transfected with siRNA against *PGRN *(Qiagen) or negative control siRNA at a final concentration of 25 nM using Lipofectamine2000 (Invitrogen). After 48 h of transfection, total RNA was extracted from SH-SY5Y cells and quantitative RT-PCR was performed as described above in the miRNA validation methods section.

### Bioinformatics analysis

It has been extensively reported that miRNAs primarily decrease mRNA expression and repress translation [38]. For the miRNA candidates significantly dysregulated in the frontal cortex and cerebellum of *PGRN+ *FTLD-TDP patients, we identified their predicted mRNA targets through TargetScan. For each of those miRNAs, we compared their predicted gene targets with mRNA expression results of *PGRN- *and *PGRN+ *FTLD-TDP patients deposited in the Gene Expression [20] database. The GEO Affymetrix array dataset published by Chen-Plotkin *et al*. (Geo record GDS3459) profiled mRNA levels in several tissue types from controls, as well as *PGRN+ *and *PGRN- *FTLD patients [39]. The significantly dysregulated miRNAs which were anti-correlated in expression with their mRNA targets in the Affymetrix data set were further analyzed by Ingenuity software for insight into their biological roles.

## Abbreviations

PGRN: progranulin; TDP-43: TAR DNA-binding protein; FTLD: frontotemporal lobar degeneration; ASTN1: astrotactin 1; PTPRD: protein tyrosine phosphatase, receptor type, D; KCNAB1: potassium voltage-gated channel, shaker-related subfamily, beta member 1; CNR1: cannabinoid receptor 1; SNCA: alpha-synuclein; NCAM1: neural cell adhesion molecule 1; BAI3: brain-specific angiogenesis inhibitor 3; GK: glycerol kinase; SLC23A2: solute carrier family 23, member 2.

## Competing interests

The authors declare that they have no competing interests.

## Authors' contributions

JK, JC and RR wrote the manuscript; JK, NK, MB, NF, and RR performed all experiments; JK, NK, KC, JC, and RR analyzed all results; JK, MB, NF, MDH, JG, DD, KAJ, NGR-R, BFB and RR categorized and prepared all patient samples. The final version of the manuscript is approved by all authors.

## Supplementary Material

Additional file 1 **miRNA analysis from *PGRN+* and *PGRN- *FTLD patients**. A table listing all results and statistical analyses from the miRNA arrays. Click here for file

Additional file 2 **Boxplot profiling of miRNAs in FTLD subtypes**. This file shows comparative boxplots for all 20 significant miRNAs identified in the miRNA arrays. Click here for file

Additional file 3 **Expression analysis of miRNAs *in vitro *with *PGRN *knockdown**. This file shows miRNA expression in human neuroblastoma SH-SY5Y cells upon siRNA knockdown of *PGRN*. Click here for file

Additional file 4 **Pathway analysis of mRNA targets for FTLD-associated miRNAs**. This file shows Ingenuity pathway analysis of anti-correlated mRNA-miRNAs in *PGRN*+ FTLD-TDP patients. Click here for file

Additional file 5 **qPCR data from miRNA validation studies**. A table listing individual Ct values in the validation data sets for both the cortex and cerebellum. Click here for file
