# Supplementary material for: Altered microRNA expression in frontotemporal lobar degeneration with TDP-43 pathology caused by progranulin mutations
Source: BMC Genomics. 2011 Oct 27;12:527. doi: 10.1186/1471-2164-12-527 (PMC3229715; doi:10.1186/1471-2164-12-527)
Supplement: Additional file 2 — Boxplot profiling of miRNAs in FTLD subtypes. This file shows comparative boxplots for all 20 significant miRNAs identified in the miRNA arrays. [file 1471-2164-12-527-S2.PDF]

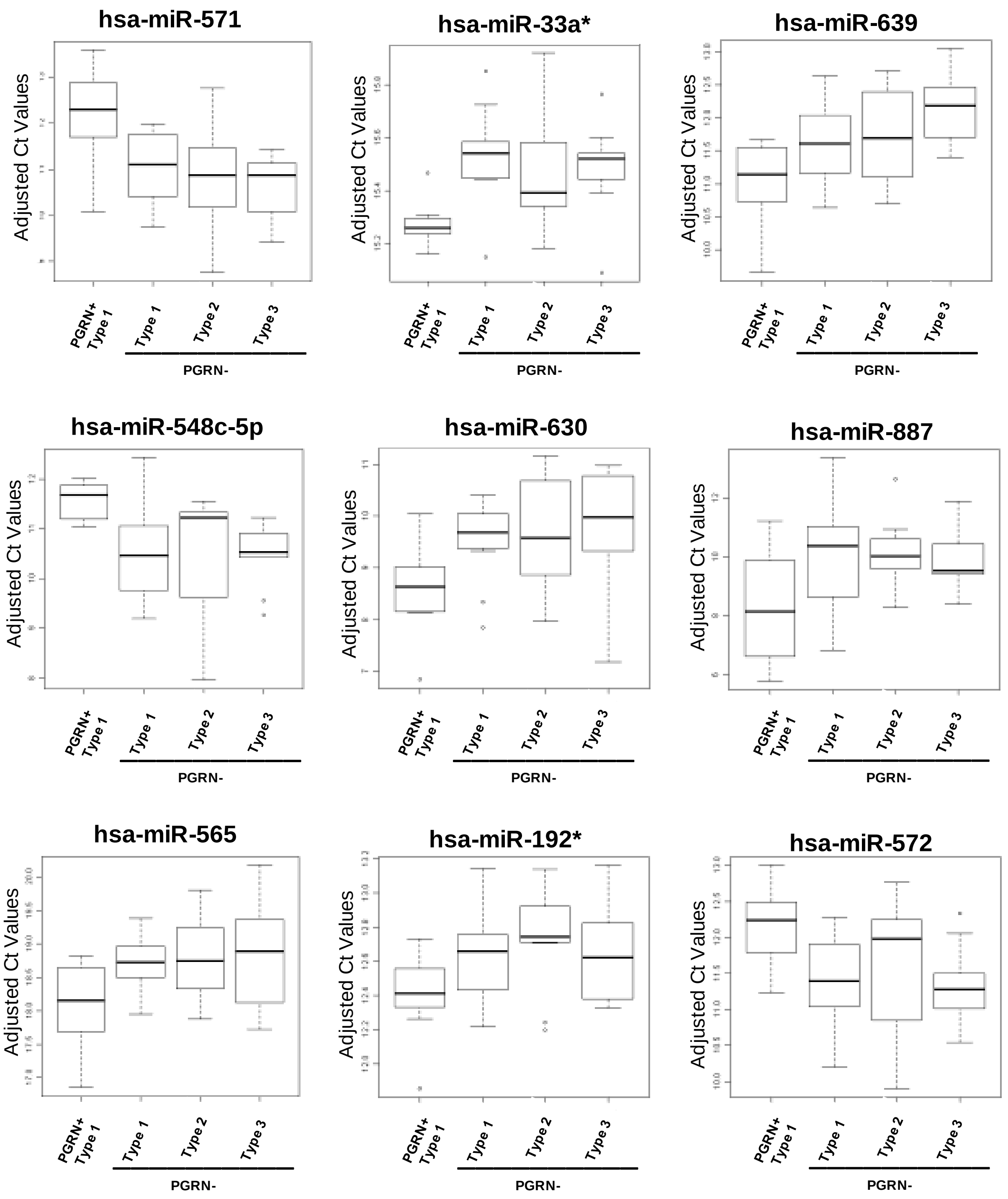
hsa-miR-571
hsa-miR-33a*
hsa-miR-639
hsa-miR-548c-5p
hsa-miR-630
hsa-miR-887
hsa-miR-565
hsa-miR-192*
hsa-miR-572
Adjusted Ct Values
PGRN+ Type 1
Type 1
Type 2
Type 3
PGRN-

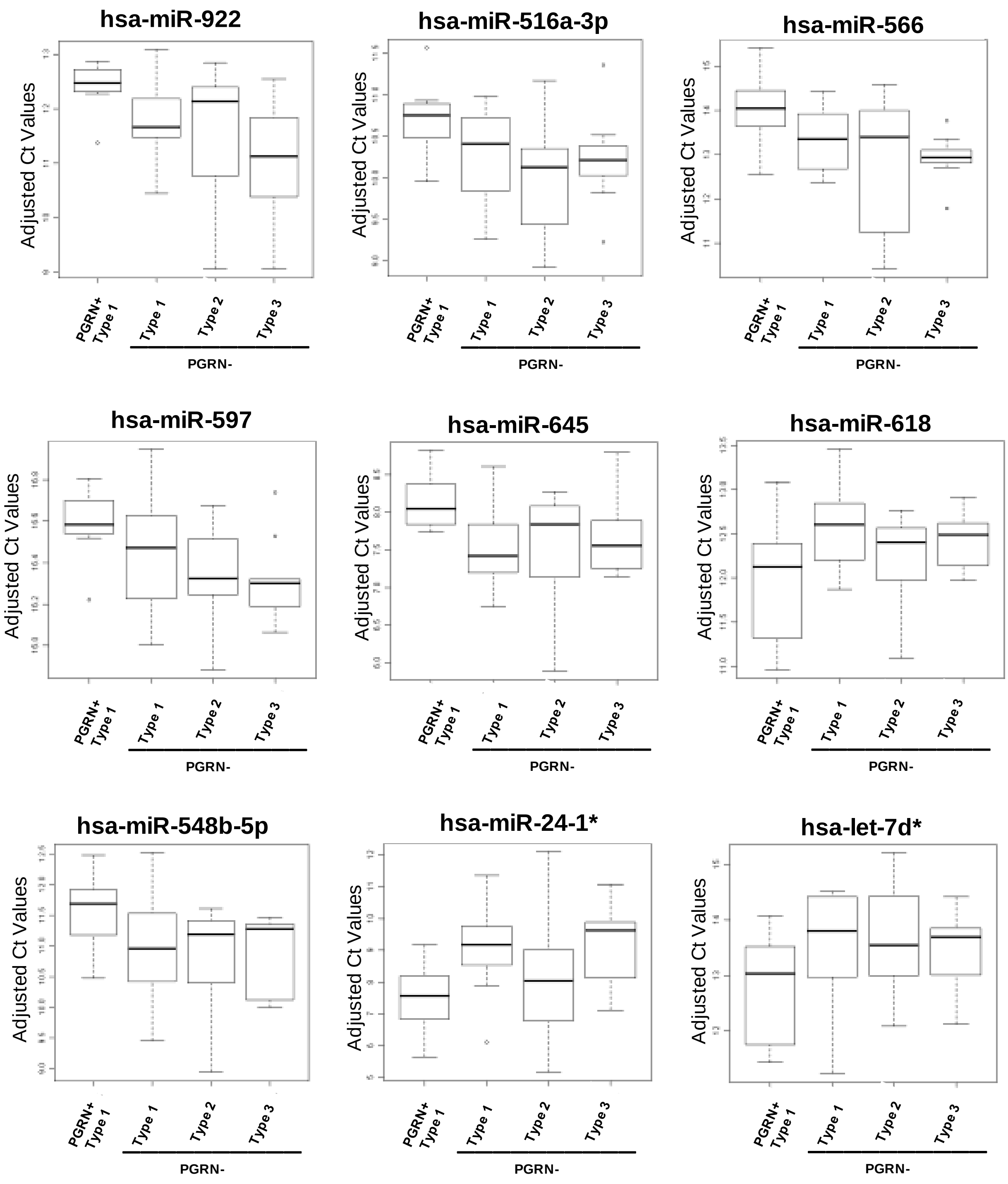
hsa-miR-922
hsa-miR-516a-3p
hsa-miR-566
hsa-miR-597
hsa-miR-645
hsa-miR-618
hsa-miR-548b-5p
hsa-miR-24-1*
hsa-let-7d*
Adjusted Ct Values
PGRN+ Type 1
Type 1
Type 2
Type 3
PGRN-

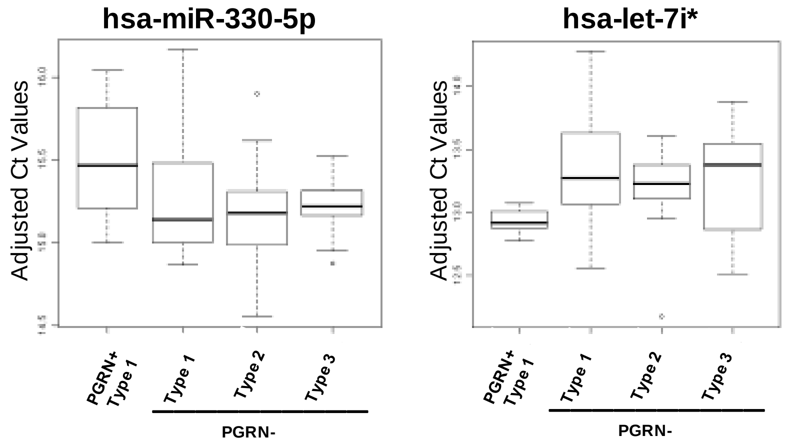

Additional file 2 - Comparative Box and Whisker plots of significant miRNAs from array profiling. Box and Whisker plots comparing miRNA expression (adjusted Ct values) between the various FTLD-TDP subtypes were generated using the built-in function in R library (R Statistical Software, Version 2.11.1). Specifically, we examined the top 20 miRNAs which were significantly dysregulated from frontal cortex tissue in the array profiling. The central line, upper and lower end of the box, represents the median, upper and lower quartile values of the miRNA expression data respectively. The bars represent the maximum and minimum values and circles denote the outlier values in the miRNA expression data. Values that are greater than 1.5 times the inter-quartile range from the upper or lower quartile were classified as outliers.
