# Supplementary material for: Altered microRNA expression in frontotemporal lobar degeneration with TDP-43 pathology caused by progranulin mutations
Source: BMC Genomics. 2011 Oct 27;12:527. doi: 10.1186/1471-2164-12-527 (PMC3229715; doi:10.1186/1471-2164-12-527)
Supplement: Additional file 3 — Expression analysis of miRNAs in vitro with PGRN knockdown. This file shows miRNA expression in human neuroblastoma SH-SY5Y cells upon siRNA knockdown of PGRN. [file 1471-2164-12-527-S3.PDF]

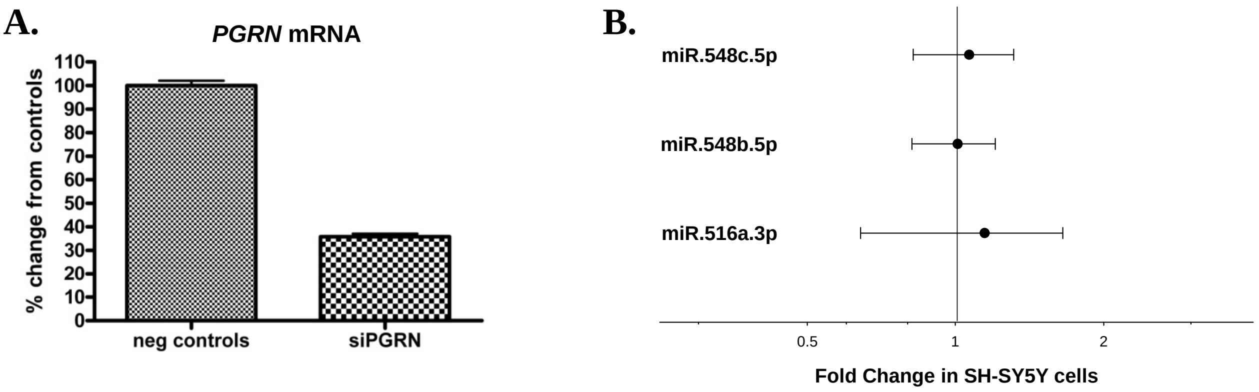

Additional file 4 – miRNA expression in human neuroblastoma SH-SY5Y cells upon siRNA knockdown of *PGRN*. Panel A). Confirmation of *PGRN* mRNA knock-down in cells transfected with *PGRN* siRNA or negative control siRNA. **Panel B).** Quantitative RT-PCR of mature miR-516a-3p, miR-548b-5p, and miR-548c-5p (total 24 for each group) fold change with 95% confidence intervals are shown for siRNA against PGRN (si-PGRN) treated cells compared to negative control siRNA treatment. RNU44 and RNU48 were used as normalization controls.
