# Supplementary material for: Altered microRNA expression in frontotemporal lobar degeneration with TDP-43 pathology caused by progranulin mutations
Source: BMC Genomics. 2011 Oct 27;12:527. doi: 10.1186/1471-2164-12-527 (PMC3229715; doi:10.1186/1471-2164-12-527)
Supplement: Additional file 4 — Pathway analysis of mRNA targets for FTLD-associated miRNAs. This file shows Ingenuity pathway analysis of anti-correlated mRNA-miRNAs in PGRN+ FTLD-TDP patients. [file 1471-2164-12-527-S4.PDF]

A.

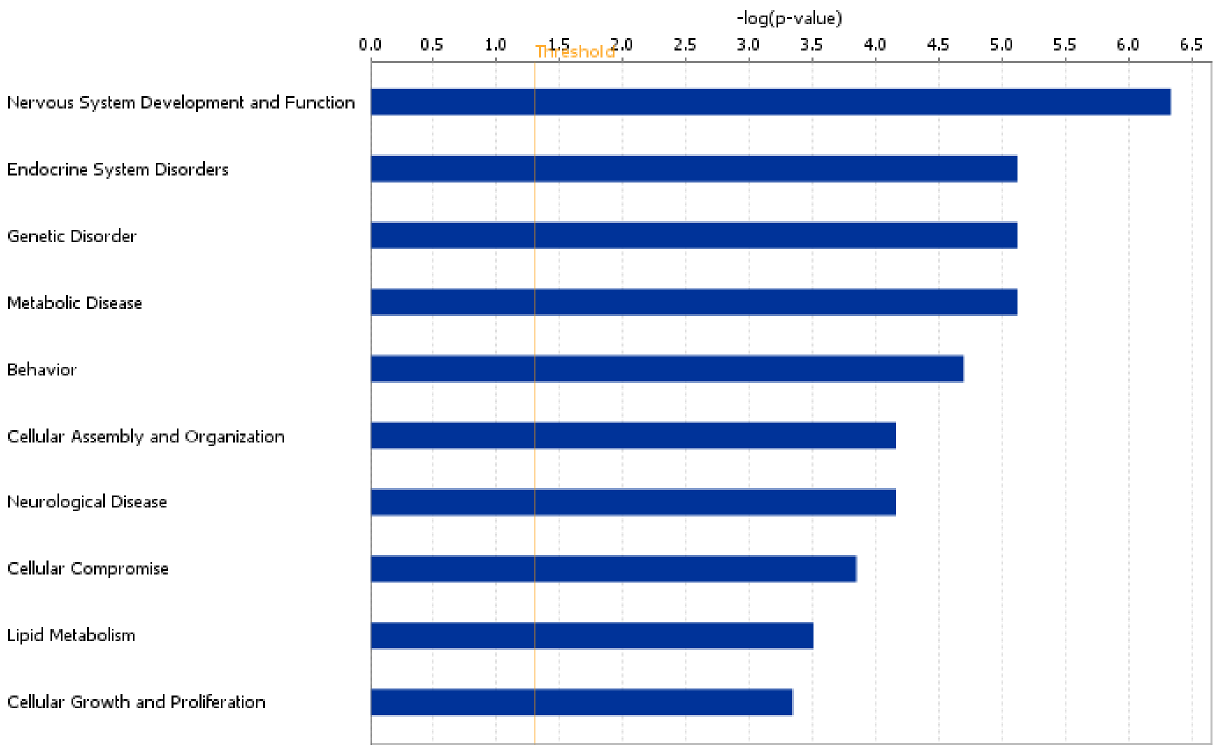

B.

Biological Function	***Genes***
Nervous System Development and Function	PTPRD, KCNAB1, FAM134B, CNR1, SNCA, NCAM1
Endocrine System Disorders	ASTN1, SLC23A2, ATP8A1, KCNAB1, CNR1, BAI3, MYT1L, TMEM135, GTDC1, NCAM1
Genetic Disorder	ATP8A1, CNR1, GK, ASTN1, SLC23A2, PTPRD, REEP1, KCNAB1, BAI3, MYT1L, TMEM135, RASA1, SNCA, GTDC1, NCAM1
Metabolic Disease	ASTN1, SLC23A2, ATP8A1, KCNAB1, CNR1, BAI3, GK, MYT1L, TMEM135, GTDC1, NCAM1
Behavior	ASTN1, PTPRD, KCNAB1, CNR1, SNCA, NCAM1
Cellular Assembly and Organization	PTPRD, CNR1, RASA1, SNCA, NCAM1
Neurological Disease	REEP1, PTPRD, KCNAB1, CNR1, BAI3, MYT1L, RASA1, SNCA, GTDC1, NCAM1
Cellular Compromise	CNR1, RASA1, SNCA, NCAM1
Lipid Metabolism	ATP8A1, CNR1, GK, SNCA, NCAM1
Cellular Growth and Proliferation	CNR1, SNCA, NCAM1

Additional data file 3 - Predicted mRNA targets (TargetScan) for the 5 miRNAs consistently dysregulated in the frontal cortex and cerebellum of *PGRN*+ FTLD-TDP patients were compared with Affymetrix mRNA data (GEO record GDS3459). A total of 18 predicted target mRNAs which were also significantly downregulated in the frontal cortex and cerebellum of *PGRN*+ FTLD-TDP patients compared to sporadic FTLD-TDP patients according to the Affymetrix results, were selected for detailed bioinformatic analyses. **Panel A).** Ingenuity pathway analysis of the 18 gene targets showing multiple significant pathways. **Panel B).** Table outlining the target genes associated with the top significant pathways identified by Ingenuity (P value < 0.05).
